# Osteoblast Differentiation on Collagen Scaffold with Immobilized Alkaline Phosphatase

**Published:** 2017-11-01

**Authors:** F. Jafary, P. Hanachi, K. Gorjipour

**Affiliations:** 1 Department of Biochemistry, School of Medicine, Shahid Sadoughi University of Medical Sciences, Yazd, Iran; 2 Faculty of Biological Science, Department of Biotechnology, Biochemistry Unit, Alzahra University, Tehran, Iran; 3 Department of Immunology, Faculty of Medicine, Shahid Beheshti University of Medical Sciences, Tehran, Iran

**Keywords:** Collagen scaffold, Alkaline phosphatase, Immobilization, Differentiation, Osteoblast

## Abstract

**Background:**

In tissue engineering, scaffold characteristics play an important role in the biological interactions between cells and the scaffold. Cell adhesion, proliferation, and activation depend on material properties used for the fabrication of scaffolds.

**Objective:**

In the present investigation, we used collagen with proper characteristics including mechanically stability, biodegradability and low antigenicity. Optimization of the scaffold was done by immobilization of alkaline phosphatase on the collagen surface via cross-linking method, because this enzyme is one of the most important markers of osteoblast, which increases inorganic phosphate concentration and promote mineralization of bone formation.

**Methods:**

Alkaline phosphatase was immobilized on a collagen surface by 1-ethyl-3-(dimethylaminopropyl) carbodiimide hydrochloride, as a reagent. Then, rat mesenchymal stem cells were cultured in osteogenic medium in control and treated groups. The osteogenesis-related genes were compared between treatments (differentiated cells with immobilized alkaline phosphatase/collagen scaffold) and control groups (differentiated cells on collagen surface without alkaline phosphatase) on days 3 and 7 by quantitative real-time PCR (QIAGEN software).

**Results:**

Several genes, including alkaline phosphatase, collagen type I and osteocalcine associated with calcium binding and mineralization, showed upregulation in expression during the first 3 days, whereas tumor necrosis factor-α, acting as an inhibitor of differentiation, was down-regulated during osteogenesis.

**Conclusion:**

Collagen scaffold with immobilized alkaline phosphatase can be utilized as a good candidate for enhancing the differentiation of osteoblasts from mesenchymal stem cells.

## INTRODUCTION

It has been shown that inorganic phosphate (P_i_) has an important role in upregulation of osteopontin (OPN) expression in MC3T3 cells [1]. Osteopontin is a phosphorylated glycoprotein secreted by osteoblasts and binds to αVβ3 integrin of cells via an RGD (arginine-glycine-aspartic acid) cell adhesion sequence [2]. It is believed that it promotes the attachment of osteoblasts and osteoclasts to the extracellular matrix. Therefore, OPN may have a key role in composition and remodeling of the bone [1]. On the other hand, β-glycerophosphate functions as a phosphate group donor in mineralization matrix, which is hydrolyzed by alkaline phosphatase. As a result, P_i _can play an important role in osteoblast differentiation and mineralization [3, 5]. 

The metalloenzyme as famous as alkaline phosphatase (ALP) [phosphate-monster phosphatase EC 3.1.3.1] is expressed in various tissues. Invertebrate ALP acts as an ectoenzyme attaching to the plasma membrane via a phosphatidyl inositol glycophospholipid (GPI) linkage. The catalytic mechanisms entail the formation of a serine phosphate at the active site, which reacts with water at an alkaline pH leading to the release of inorganic phosphate from the enzyme. Four ALP isozymes—intestinal, placental, placental-like, and tissue non-specific ALP (TNAP)—are available in human. TNAP is an osteoblastic differentiation marker that is expressed in the initial phases of the process. In addition, the enzyme is necessary for mineralization of bone. Furthermore, the greater ALP activity, the great impact it might have on bone formation. Accordingly, ALP has a critical role in bone formation [6].

Many factors such as pore size, porosity, permeability, and the composition of the scaffold have been indicated to regulate osteoblasts behavior [7]. Collagen is one of the critical proteins in the extracellular matrix of numerous connective tissues. Thus far, different types of collagen (approximately 29) have been identified. The most abundant type of collagen in the body is type I and is found in bones, skins, tendons, vessels, and organs. The structural, physical, chemical, and immunological properties of collagen have been identified, which include its biodegradability and biocompatibility, being non-cytotoxic, and having the ability to support cellular growth. Indeed, the collagen can be processed into various forms such as steps, sheets, beads, meshes, fibers, and sponges. Type I collagen can develop mineral to matrix ratio, calcium deposition and osteopontin/osteocalcin secretion [8]. These data suggest that collagen has the most important role in promoting osteogenesis and could be an efficient scaffold to be used in bone tissue engineering.

This study aimed to develop a method for immobilization of ALP on collagen fibers scaffolds using covalent bond and 1-ethyl-3-(dimethylaminopropyl) carbodiimide hydrochloride (EDC) as cross-linkers. Afterwards, the ability of creating scaffolds in mineralization and osteoblast differentiation were characterized *in vitro*.

## MATERIALS AND METHODS

All reagents used in this experimental study were supplied by Sigma (CA, USA) unless indicated otherwise. This study was conducted on human bone marrow-derived mesenchymal stem cells (MSCs) that were differentiated on a collagen scaffold affected by alkaline phosphatase. Various parts of the methods used were approved by Iran National Science Foundation (grant number 90004378).

Activation of the Collagen Fiber and Immobilization of the Enzyme

The collagen matrix (Viscofan cell carrier) was treated with 10 mg/mL of 1-ethyl-3-(dimethylaminopropyl) carbodiimide hydrochloride (EDC) (Piers 22980) in phosphate buffer for 30 min [9]. Subsequently, the matrix was washed with PBS and incubated in aqueous bovine intestinal mucosa alkaline phosphatase (3 U) (Sigma P6774) for 2 hrs. The collagen matrix was rinsed in PBS four times each for 15 min with shaking.

The Alkaline Phosphatase Activity Assay

The enzyme activity was detected by Taylor, *et al*, method (2006). The p-nitrophenylphosphate (pNPP) (Sigma P4744) was selected as the substrate [10]. A stock solution of pump was 350 µM in 25 mm glycine with pH 9.5. The collagen matrix with immobilized enzyme was treated with 2 mL of substrate at room temperature. Then, every minute, 90 µL of 0.1 M NaOH and 0.1 M EDTA was added as a stop solution and finally the absorbance was measured at 405 nm. The alkaline phosphatase activity was calculated using the method of Taylor at al, 2005, and the following equation:

Stem Cell Isolation and Differentiation

MSCs were isolated from adipose tissue of three patients who referred to Shahid Sadughi Hospital for abdominal surgery. Adipose tissue was digested by enzymatic method (1 mg collagenase per 1 g adipose tissue). The isolated cells were then cultured in culture medium containing DMEM, 10% fetal bovine serum, 1% penicillin/streptomycin (Gibco) at 37 °C, and 5% CO_2_. Cells in the third passage were added to osteogenic medium, including the above-mentioned culture medium and 10 mM β-glycerol phosphate, 50 µg/mL ascorbic acid, and 10^-8^ M dexamethasone. This medium was used for differentiation of approximately 10^5^ cells into two groups: the treatment group (in presence of ALP/collagen scaffold) and the control group (in the absence of the ALP/collagen scaffold).

Total RNA Extraction and Complementary DNA Synthesis

The total RNA was isolated from osteoblasts differentiated from MSCs on days 3 and 7 using RNeasy Mini Kit Qiagen according to the manufacturer’s protocol in the treatment group (in presence of the ALP/collagen scaffold) and the control group (in the absence of the ALP/collagen scaffold). The concentration of RNA was determined at 260/280 nm using NanoDrop spectrophotometer. For reverse transcription polymerase chain reaction (RT-PCR) the cDNA was synthesized by RevertAid First Strand cDNA Synthesis (Thermo Scientific) following the instructions provided. The synthesized cDNA was stored at 20 °C for later use.

Real-time RT-PCR

Simultaneous gene expression level for OCN.COL, TNF, ALP and Runx2 genes along with β-actin house-keeping gene were measured by three steps real-time PCR (Corbett research, Sydney, Australia) using SYBR green method. The primers used for PCR were as follows:

Runx2:

Forward:

5’ CTTCATTTGCACTGGGTCAC 3’ (Tm: 58.4)

Reverse: 

5’ CTGCAAATCTCAGCCATGTT 3’ (Tm: 56.4)

Type I collagen:

Forward: 

5’ ACCAACTGAACGTGACCAAA 3’ (Tm: 56.4)

Reverse:

5’ AGTGGGCAGAAAGGGACTTA 3’ (Tm: 58.4)

-actin:

Forward: 

5’ GCTGTGCTATGTTGCCCTAGA 3’ (Tm: 64.0)

Reverse: 

5’ GGAACCGCTCATTGCCGATAG 3’ (Tm: 63.3)

Alkaline phosphatase:

Forward:

5’ CGTCAATTAACGGCTGACAC 3’ (Tm: 58.4)

Reverse: 

5’ TCTGGCACAAATGAGTTGGT 3’ (Tm: 56.4)

TNF:

Forward: 

5’ CTGTGCACTCCTGGTGTTCT 3’ (Tm: 60.5)

Reverse:

5’ TGTCTTCCTCCTGACTGTGC 3’ (Tm: 60.5)

And, Osteocalcin:

Forward:

5’ CTGCATTCTGCCTCTCTGAC 3’ (Tm: 60.5)

Reverse: 

5’ CCGGAGTCTATTCACCACCT 3’ (Tm: 60.5).

Real-time PCR reaction was carried out in 20 µL volume including Taq PCR Master Mix 10 µL, forward and reverse primers (0.5 µL for each), 1 µL of cDNA, and 8 µL of dH_2_O according to cycling program (Table 1).

**Table 1:** Cycling program temperature and time

Phase	Duration	Temperature °C
Hold	15 min	95
PCR initial denaturation step	10 sec	94
Denaturation	25 sec	94
Annealing & Extension	30 sec	72

The analysis of the results was performed using REST RG (Relative Expression Software Tool) software that enables more sensitive and accurate estimation of the relative gene expression.

## RESULTS

Properties of the Immobilized ALP

**Figure 1:**
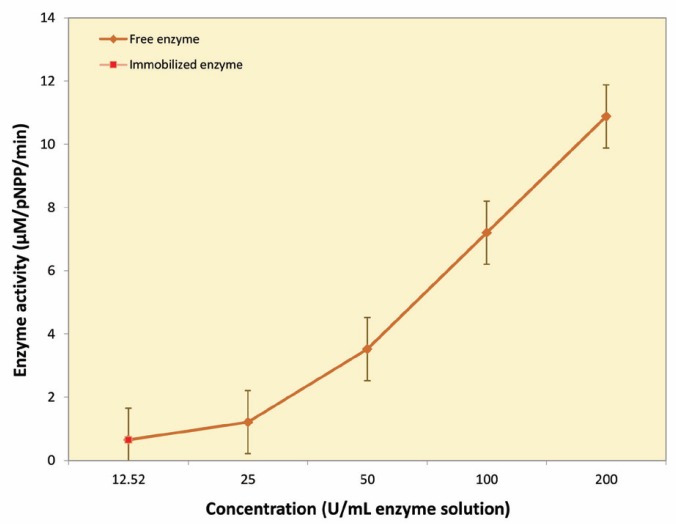
The standard activity curve. This curve estimates the amount of the immobilized enzyme

Alkaline phosphatase was covalently immobilized on collagen matrix by 1-ethyl-3-(dimethylaminopropyl) carbodiimide hydrochloride (EDC) as reagent. The optimum pH and temperature, thermal stability, reusability, and storage stability of the immobilized enzyme were studied in a previous study [11]. Our results showed that the immobilization was done successfully and that the physiochemical properties of the ALP could be improved. The size of collagen matrix was 4.5 cm^2^; the total amount of the immobilized enzyme on the scaffold was measured in 12 units (Fig 1).

Quantitative Real-time PCR

**Figure 2:**
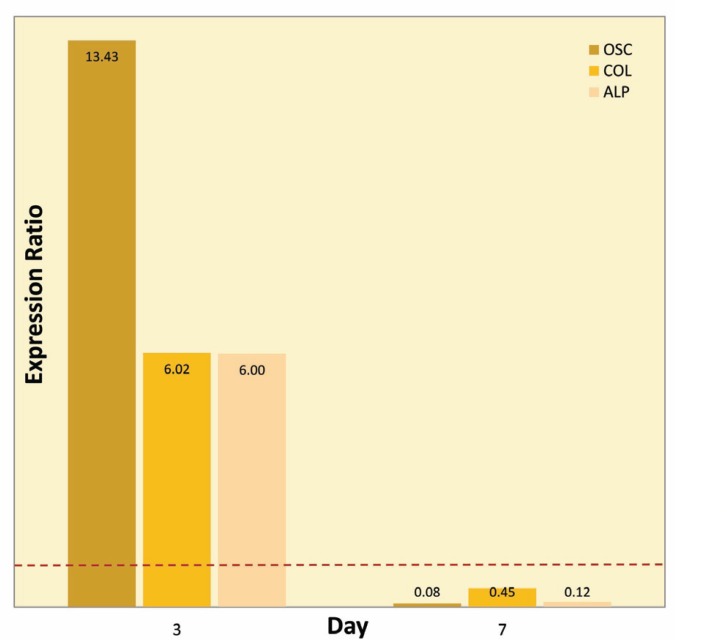
Relative gene expression for osteocalcin, alkaline phosphatase, and collagen I; the three markers levels in treated groups on the 3^rd^ day were more than those on the 7^th^ day in comparison to the control group. Dashed red line represents a ratio of 1

**Figure 3:**
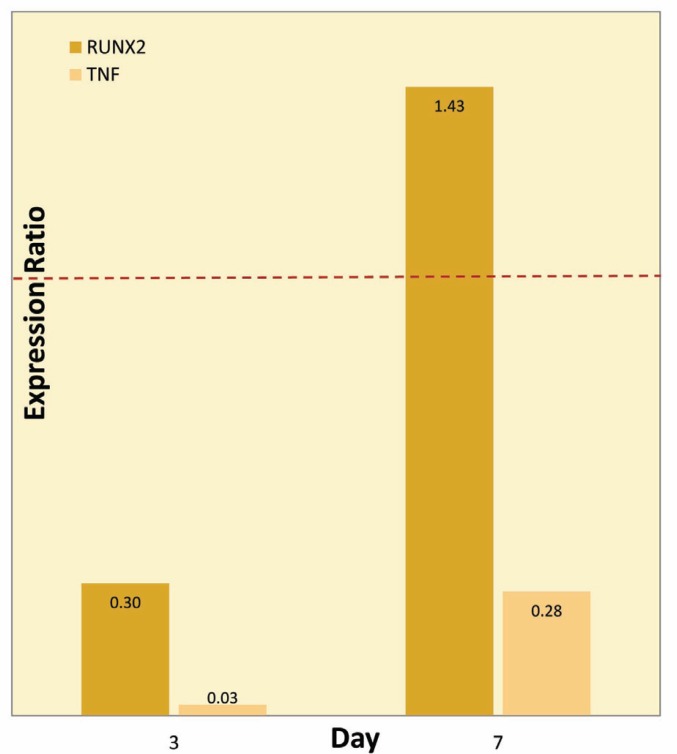
Relative gene expression for Runx2 and TNF-α; the two markers were down-regulated on the 3^rd^ day in the treated groups in comparison to those on the 7^th^ day. Dashed red line represents a ratio of 1.

Expression analysis of osteogenesis-related genes in the treated and control groups on the 3^rd^ and 7^th^ days are presented in Figures 2 and 3. The amplification sufficiency for all markers ranged between 0.8 and 1.9. This result indicated the PCR was optimized. 

Osteocalcin, Alkaline Phosphatase, and Type I Collagen Expression

Real-time PCR analysis revealed a significant (p<0.001) difference in the three markers levels in treated groups on the 3^rd^ day. Osteocalcine (OSC), alkaline phosphatase (ALP) and collagen I (col I) were up-regulated in the treated groups in comparison to the control group (Fig 2). However, these genes expressions decreased significantly (p<0. 001) on the 7^th^ day. OSN, ALP, and col I were down-regulated in the treated groups in comparison to the control group respectively (Fig 2). 

Runx2 and TNF-α Expression

Runx2 and TNF-α are two critical regulator factors of osteoblast differentiation. Their expressions decrease during maturation of osteoblast. Real-time PCR showed that these two markers were down-regulated in the treated groups in comparison to the control group on the 3^rd^ day (Fig 3) However, there was no significant difference in the expression of these genes in the treated and control groups (p=0.506).

## DISCUSSION

In the present study, we used collagen as a scaffold for enzyme immobilization and osteoblast differentiation. Previously, collagen has been applied as a scaffold in biological systems [12]. The high tensile strength, mechanical stability, and biodegradability of collagen are of great values in biomedical and tissue engineering [7]. ALP was immobilized on the collagen surface because the collagen has appropriate chemical groups such as carboxyl groups, which can participate in cross-linking reactions. About 12 units of enzymes were immobilized on the matrix (4.5 cm^2^) during the process. The results showed that collagen matrix had a satisfactory capability to preserve enzyme and also its activity (48.6%) [11]. 

Collagen is also an appropriate matrix for proliferation, differentiation, and attachment of different kinds of cells. Various types of collagen may have a critical role in cell behavior depending on the organ or tissue of origin [13, 15]. In this study, type I collagen, the critical protein component of bone, was used for osteoblast differentiation. Stem cells interaction with type I collagen via α2β1 integrin, which is a major signal for the induction of osteoblast differentiation and matrix mineralization [16, 17]. Integrin is a trans-membrane receptor that has an important role in cell interaction with other cells and the surrounding environment. Indeed, α2β1 integrin specific collagen-mimetic surface was reported to supports osteoblastic differentiation [18]. According to the obtained results, ALP immobilization onto type I collagen stimulated *in vitro* differentiation of osteoblasts from MSCs *in vitro*.

Immobilization of ALP on collagen leads to an increase in the concentration of P_i_ in cell culture from hydrolysis of β-glycerophosphate. Previous studies showed that modeling the local concentration of P_i_ can be useful for bone formation [19].

Different steps of osteoblast differentiation process including proliferative, extracellular matrix synthesis, and mineralization are detected by expression of specific genes. Our quantitative Real-time PCR showed induction of the expression of osteoblast markers mRNA through increased concentration of P_i_ that explained as follows: ALP enzyme attached to membrane phospholipids of the matrix vesicles. Therefore, ALP activity was increased during the matrix maturation phase. In fact, ALP is considered one of the most commonly accessible marker to indicate osteoblast differentiation [20]. In a previous study, we reported that ALP increased during osteogenesis up to three weeks [21]. George, *et al*, investigated differentiation of mesenchymal stem cells into osteoblast on the honeycomb collagen scaffold. In their research, ALP activity was increased to about three folds on day 28 compared with day 14 [12]. ALP was immobilized on the microporous nanofibrous fibrin scaffold for bone tissue engineering by Osathanon, *et al.* They showed that ALP/FS scaffold can be helpful in osteogenesis. The expression of ALP was elevated on the 7^th^ and 14^th^ days compared to the control group [19]. In our study, the expression of ALP was up-regulated at the 3^rd^ day of differentiation and was decreased within 7 days. This result indicated that maturation of the matrix was started as early as three days in the treated cells. 

Osteocalcin, also known as bone g-carboxyglutamic acid-containing protein, is a small noncollagenous protein synthesized by osteoblasts in the final stage of osteogenic maturation. OSN has an important role as a regulator of mineral nucleation via bind to hydroxyapatite. The increased expression of OCN, therefore, shows maturation of osteoblasts will occur. Osteocalcine gene expression was detected at the second week by Ducy, *et al* [22]. The expression of OCN was reported on the 21^st^ day in the study performed by Osathanon, *et al *[19]. In our research, OCN was up-regulated in the treated groups in comparison to the control group on the 3^rd^ day. However, it was down-regulated on the 7^th^ day. The results could show a high rate of differentiation on ALP/collagen scaffold.

The primary function of differentiated osteoblasts is collagen synthesis. So collagen is a specific gene of early- and mid-stage of osteogenesis. COL with unique triple helical structure, plays an important role in the matrix maturation. This protein acts as a substrate for precipitation of hydroxyapatite crystals. Therefore, collagen is an essential component and provides mechanical stability for bone [23]. Osathanon, *et al*, reported increased collagen expression on the 7^th^ day; the expression decreased on the 21^st^ day. In our research, COL was up-regulated on the 3^rd^ day and declined thereafter [19]. COL is a marker of osteoblast differentiation that elevates in proliferative and matrix maturation phases. Immobilized ALP/collagen scaffold showed significant fold increases in osteogenic markers on day three compared to that on day seven, which suggested its high proliferation and differentiation.

Runt-related transcription factor 2 (Runx2) or osteoblast-specific factor (Osf2) that has been named “a master gene,” is an essential transcription factor for the initiation of osteogenesis. Several osteoblastic gene expressions, including collagen I, osteopontin and bone sialo protein are regulated by Runx2 at early stages of osteoblast differentiation. The level of Runx2 expression in immature osteoblasts is high, but it will be down-regulated in immature osteoblasts during osteogenesis. Therefore, Runx2 plays an essential role in inducing differentiation of multipotent MSCs into immature osteoblasts. However, osteoblasts differentiation and bone formation are inhibited by Runx2. The expression of a critical bone matrix gene depends on Runx2 in early stages of osteoblast differentiation, but it has no effect on the maintenance of the expression of these genes in mature osteoblasts. Normally, the expression level of Runx2 in osteoblasts declines during differentiation process [24]. Hernan Roca, *et al*, analyzed the Runx2-deficiency in mice. They reported complete lack of mature osteoblasts with only a few immature osteoblasts, which expressed ALP weakly, but not OPN and osteocalcin (OCN) [25]. 

In our research, an expression level of Runx2 in cells on the 3^rd^ day was down-regulated in the treated groups in comparison to the control group. There was no significant difference between treated and control groups on the 7^th^ day.

TNF-α (also known as cachectin) has been identified as a pro-inflammatory cytokine that plays a role in many skeletal diseases. TNF-α arrests expression of osteoblast differentiation markers such as Runx2, osteocalcin, and ALP. Indeed, inhibition of collagen and osteocalcin synthesis leads to reduced formation of mineralizing nodules and the skeletal matrix. Accordingly, TNF-α decreases bone formation [26]. In our research, TNF-α expression decreased on day three in the treated group. However, no significant difference in expression of this gene was observed between the treated and control groups.

Previous studies demonstrated that collagen can be a perfect scaffold for the differentiation and proliferation of MSCs into osteoblast. Collagen with its several properties like low antigenicity, biodegradability, and excellent hemostatic and cell binding can be applied in medical and biomedical industries extensively [27, 28]. However, collagen with ALP immobilized has previously not been used as a scaffold. In accord with our result, immobilization of ALP onto collagen, makes a suitable scaffold for enhancing osteoblast differentiation from MSCs. The expression of genes associated with the final mineralization phase such as OSC was observed on the 3^rd^ day., A previous study showed similar results in the third week. Indeed, the expression of the inhibitor gene for osteoblast differentiation was decreased during differentiation. Interestingly, osteogenesis was dedifferentiated within seven days. Perhaps this result is related to overexpression of the osteopontine effect of phosphate signaling, because phosphate is a signal for induction of osteopontin gene expression [1]. Huang,* et al, *found that osteopontin is a negative regulator of proliferation and differentiation in MC3T3-E1 pre-osteoblastic cells. They reported that overexpression of OPN inhibits the effect of bone morphogenic protein-2 (BMP-2) on ALP activity. In addition, overexpression of OPN inhibits mineral deposition and also decreases the expression of osteocalcin and bone sialoproteins. As a result, ALP immobilized causes an increase in the level of P_i_ and can lead to increasing OPN expression.

In conclusion, collagen characteristics such as having appropriate groups, being mechanically stable, and its biodegradability, low antigenicity and cell binding makes type I collagen an excellent candidate for enzyme immobilization and also cell culture. ALP is a critical molecule in mineralization of bone formation and its immobilization on a collagen scaffold leads to increase in the concentration of P_i_ in cell culture. Our results showed that the collagen with immobilized ALP has a positive effect on osteoblast differentiation and via up-regulation of osteogenesis-related genes expression such as ALP, COL, OCN, and down-regulation of inhibitor genes expression like TNF-α. In summary, our results propose that the applied immobilized ALP/collagen matrix is more effective than collagen alone for increasing the rate of osteoblast differentiation. 
